# Recurrent Spontaneous Chylopericardium Treated by Thoracic Duct Embolization

**DOI:** 10.1016/j.jaccas.2026.108162

**Published:** 2026-05-05

**Authors:** David Bobrowski, Dheeraj Rajan, Cathal O'Leary, Jeremy Edwards

**Affiliations:** a Department of Medicine, University of Toronto, Toronto, Ontario, Canada; b Division of Vascular & Interventional Radiology, University Medical Imaging Toronto, Joint Department of Medical Imaging, University Health Network, Mount Sinai Hospital and Women's College Hospital, University of Toronto, Toronto General Hospital, Toronto, Ontario, Canada; c Department of Medical Imaging, University of Toronto, Toronto, Ontario, Canada; d Division of Cardiology, St Michael's Hospital, Toronto, Ontario, Canada

**Keywords:** echocardiography, imaging, pericardial effusion, tamponade, thoracic

## Abstract

**Background:**

Spontaneous chylopericardium is a rare etiology of pericardial effusion and is a diagnosis of exclusion.

**Case Summary:**

A 45-year-old woman was incidentally found to have a large pericardial effusion on abdominal ultrasound performed for mildly elevated liver enzymes. Transthoracic echocardiography demonstrated early tamponade physiology, prompting urgent pericardiocentesis with drainage of chylous fluid. After early recurrence, intranodal magnetic resonance lymphangiography demonstrated reflux, and catheter-based lymphangiography confirmed a leak arising from the thoracic duct. She underwent percutaneous thoracic duct embolization with concomitant pericardial drainage, resulting in complete resolution without recurrence on follow-up.

**Discussion:**

Magnetic resonance lymphangiography can identify occult sources of chylopericardium. Percutaneous thoracic duct embolization provides a minimally invasive alternative to surgical ligation for definitive treatment of thoracic duct leaks.

**Take-Home Messages:**

Recurrent spontaneous chylopericardium should prompt evaluation for thoracic duct pathology. Potentially curative thoracic duct embolization should be considered early.

## Visual Summary

**Figure:**
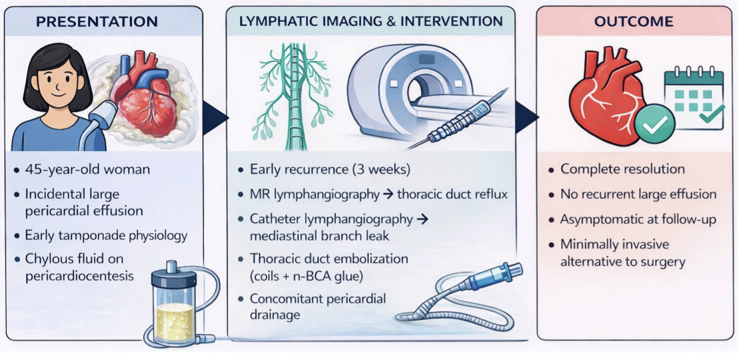

**Visual Summary:**
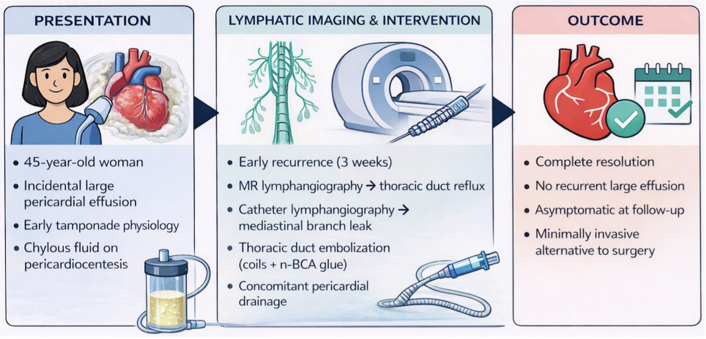
Recurrent Spontaneous Chylopericardium Treated by Thoracic Duct Embolization MR = magnetic resonance.

## Case Presentation

A previously healthy 45-year-old woman was referred to the emergency department after an outpatient abdominal ultrasound, performed for mildly elevated liver enzymes, incidentally revealed a large pericardial effusion. She was asymptomatic and hemodynamically stable on presentation.

**Take-Home Messages**

• Recurrent spontaneous chylopericardium should prompt evaluation for thoracic duct pathology.
• Potentially curative thoracic duct embolization should be considered early.

**Figure 1:**
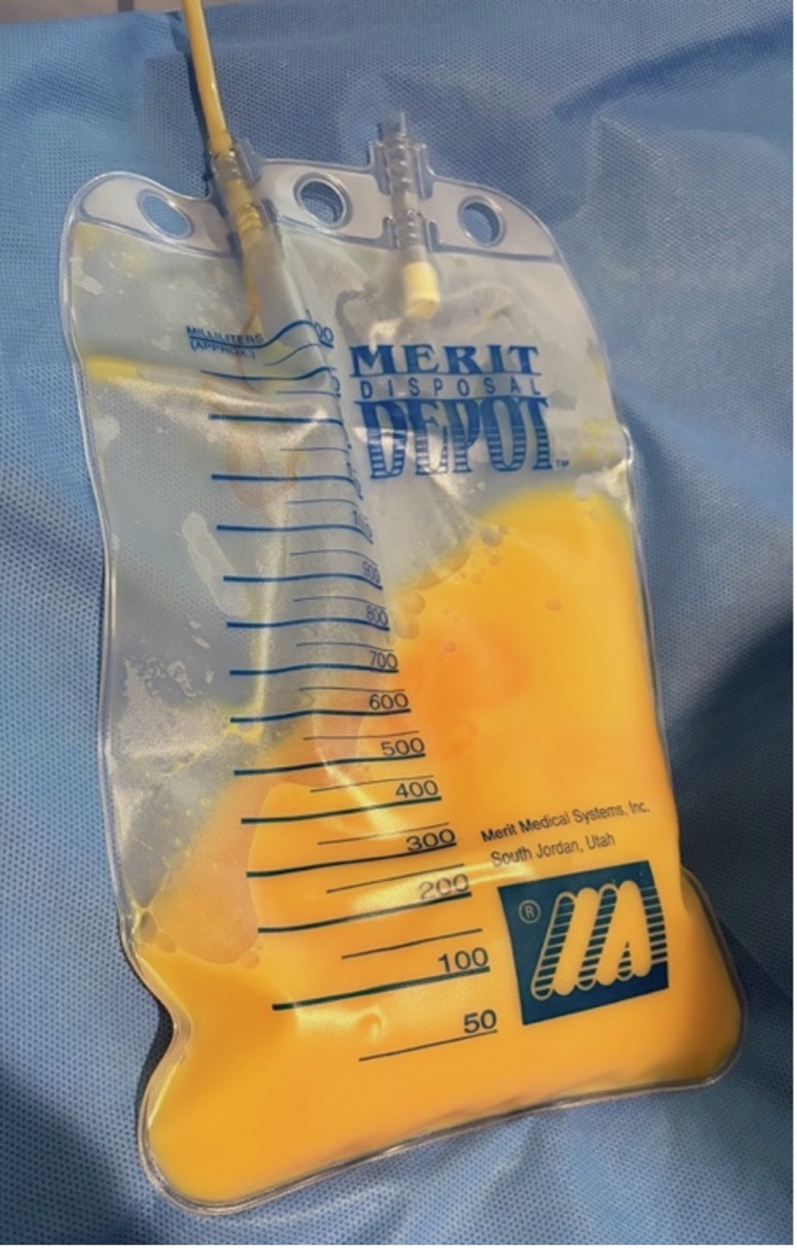
Chylous Pericardial Fluid Chylous fluid drained from the pericardial space after pericardiocentesis.

**Figure 2:**
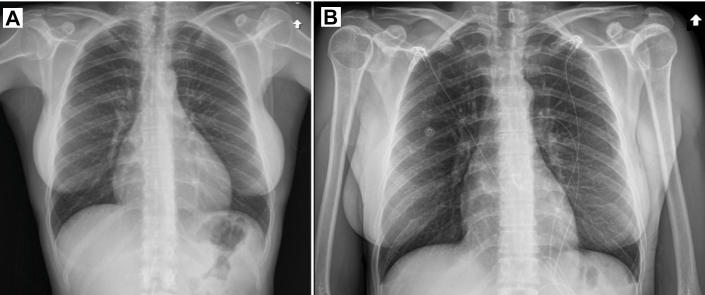
Chest Radiographs Before and After Pericardiocentesis Upright anteroposterior chest radiographs (A) before and (B) after pericardiocentesis. Compared with the initial study, the postprocedural image demonstrates a reduction in the cardiopericardial silhouette after removal of the pericardial drain.

Transthoracic echocardiography (TTE) demonstrated a large circumferential pericardial effusion measuring up to approximately 4 cm with signs of tamponade, including right ventricular diastolic collapse and a dilated, noncollapsing inferior vena cava (Video 1). Given these findings, urgent diagnostic and therapeutic pericardiocentesis was performed, draining approximately 350 mL of yellow and turbid fluid (Figure 1). Pericardial fluid analysis demonstrated lymphocyte predominance with markedly elevated triglycerides and low cholesterol, consistent with chylopericardium. Bacterial, fungal, and mycobacterial cultures were negative, and cytology showed no evidence of malignancy. Computed tomography (CT) of the chest, abdomen, and pelvis showed no evidence of lymphadenopathy, mediastinal neoplasms, central vein thrombosis, or other structural cause of the effusion. Laboratory evaluation, including rheumatologic testing, viral studies, and cardiac biomarkers, was unrevealing. The results of laboratory testing for secondary causes are summarized in Table 1. There were no historical or physical examination features of trauma or autoimmune disease. Postprocedural echocardiography verified only trace residual effusion, and chest x-ray showed a smaller cardiopericardial silhouette (Figures 2A and 2B).

**Table 1:** Results of Laboratory Investigations

	Result	Reference Range
Hemoglobin	145 g/L	115-155 g/L
Leukocyte count	7.8 × 10^9^/L	4.0-11.0 × 10^9^/L
Platelet count	210 × 10^9^/L	140-400 × 10^9^/L
Creatinine	77 μmol/L	49-90 μmol/L
Troponin I	3 ng/L	0-12 ng/L
International normalized ratio	1.2	0.9-1.2
Aspartate aminotransferase	40 U/L	13-39 U/L
Alanine aminotransferase	63 U/L	7-52 U/L
Alkaline phosphatase	84 U/L	34-104 U/L
Bilirubin	8 μmol/L	0-17 μmol/L
Lipase	14 U/L	<60 U/L
C-reactive protein	0.9 mg/L	0.0-5.0 mg/L
Erythrocyte sedimentation rate	10 mm/h	20 mm/h
Nuclear antibody	Negative	N/A
DNA double-stranded antibody	<10 IU/mL	≤99 IU/mL
Myeloperoxidase antibody	4 RU/mL	<20 RU/mL
Proteinase 3 antibody	<2 RU/mL	<20 RU/mL
Complement C3	0.93 g/L	0.87-2.00 g/L
Complement C4	0.26 g/L	0.19-0.52 g/L
Cytomegalovirus antibody IgM	Nonreactive	N/A
COVID-19, RSV, influenza A/B nasopharyngeal swab PCR	Negative	N/A
Cytomegalovirus antibody IgG	Reactive	N/A
Epstein-Barr virus capsid antibody IgM	Nonreactive	N/A
Epstein-Barr virus capsid antibody IgG	Reactive	N/A
Epstein-Barr virus nuclear antibody IgG	Reactive	N/A
Hepatitis A virus antibody IgM	Nonreactive	N/A
Hepatitis A virus antibody IgG	Reactive	N/A
Hepatitis B virus surface antibody	2.0 IU/L	N/A
Hepatitis B virus surface antigen	Nonreactive	N/A
Hepatitis B virus core antibody	Nonreactive	N/A
Hepatitis C virus antibody	Nonreactive	N/A
Triglycerides	0.83 mmol/L	<2 mmol/L
Cholesterol	5.4 mmol/L	<5.2 mmol/L
Triglycerides in pericardial fluid	20.15 mmol/L	N/A
Cholesterol in pericardial fluid	2.64 mmol/L	N/A
Chylomicrons in pericardial fluid	Present	N/A
Lactate dehydrogenase in pericardial fluid	111 U/L	N/A
Protein in pericardial fluid	47 g/L	N/A
Glucose in pericardial fluid	5.4 mmol/L	N/A
pH of pericardial fluid	7.65	N/A
Cell count in pericardial fluid	96.1% lymphocytes (1,274 × 10^6^/L leukocytes)	N/A
Gram stain/culture of pericardial fluid	Negative	N/A
Acid-fast bacilli smear	Negative	N/A
Mycobacteria culture of pericardial fluid	Negative after 7 wk	N/A
Cytology of pericardial fluid	Negative for malignancy	N/A

Secondary work-up, including infectious serologies, autoimmune and rheumatologic markers, hepatobiliary indices, and pericardial fluid cytology, was negative. Pericardial fluid triglycerides were markedly elevated with the presence of chylomicrons.

N/A = not applicable; PCR = polymerase chain reaction; RSV = respiratory syncytial virus.

**Figure 3:**
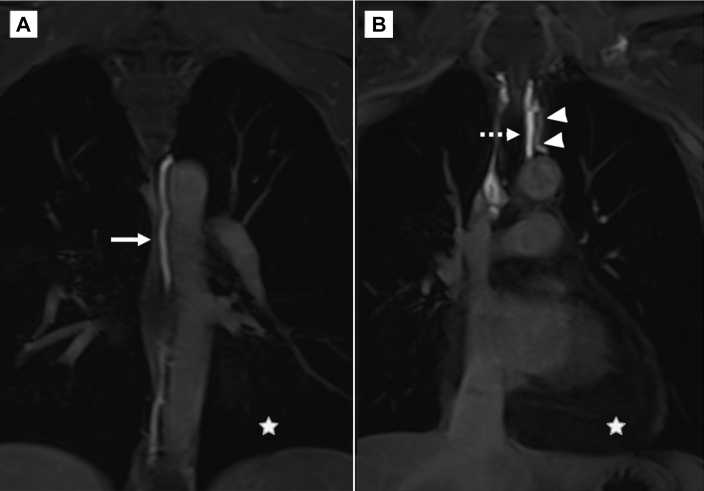
Magnetic Resonance Lymphangiogram With Postcontrast Fat-Suppressed Acquisition in the Coronal Plane (A) Single thoracic duct from the cisterna chyli to the aortic arch (white arrow). Large pericardial effusion is present (white star). (B) Superior thoracic duct (dashed white arrow) with reflux in the left anterior mediastinal branch (white arrowheads). A solitary terminal thoracic duct and lymphovenous junction was identified (not shown).

**Figure 4:**
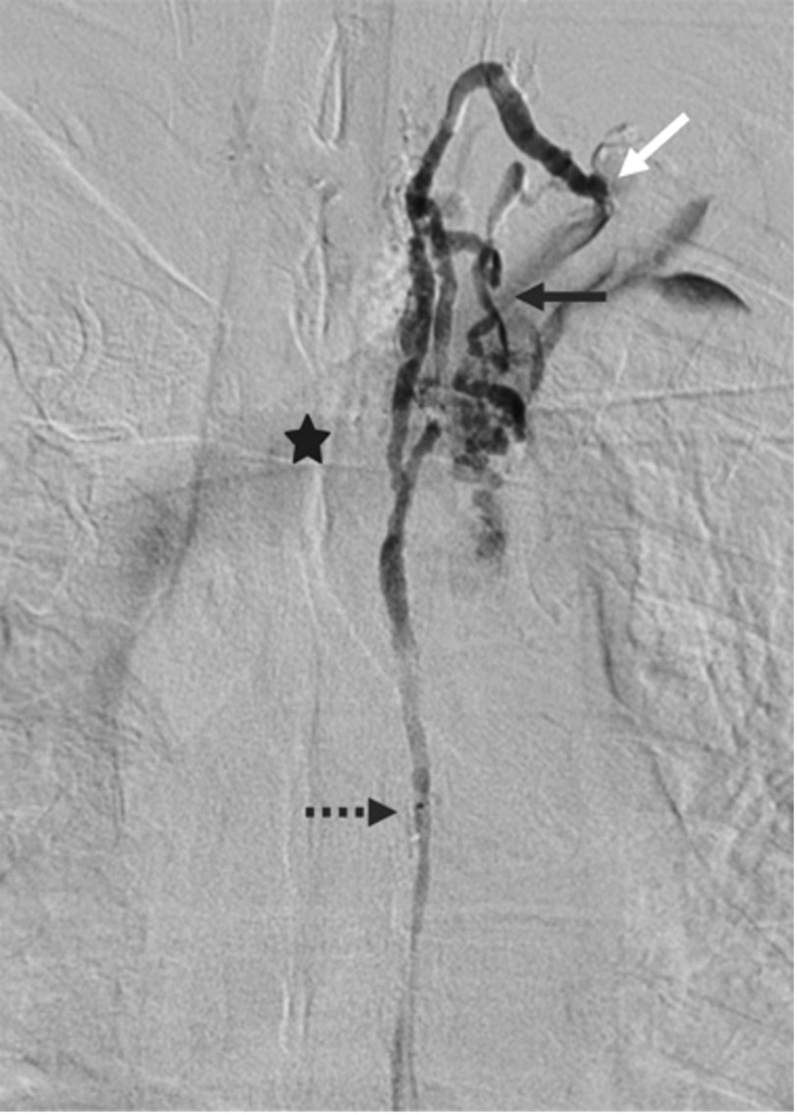
Catheter Lymphangiogram Digital subtraction lymphangiogram via a microcatheter in the midthoracic duct at the level of the carina (dashed arrow). Iodinated contrast flows through the lymphovenous junction (white arrow) into the brachiocephalic vein (black star). The source of the leak is reflux in the left anterior mediastinal branch (black arrow). Contrast injection from catheter-based lymphangiography demonstrated a patent thoracic duct with reflux into a left anterior mediastinal branch, extending into the mediastinum and corresponding to the suspected pericardial leak seen on magnetic resonance imaging.

Three weeks later, surveillance echocardiography revealed reaccumulation of a large pericardial effusion, although the patient remained asymptomatic and without tamponade physiology. Intranodal contrast-enhanced magnetic resonance (MR) lymphangiography demonstrated a solitary lower midthoracic duct with reflux within a tributary of the superior thoracic duct, raising concern for a thoracic duct leak (Figures 3A and 3B). Subsequent catheter-based lymphangiography demonstrated a single thoracic duct and a patent lymphovenous junction, with a leak arising from reflux in the left anterior mediastinal branch, providing an anatomic explanation for the recurrent chylopericardium (Figure 4). The thoracic duct was embolized with 4- to 6-mm Nester coils (Cook Medical) and n-BCA glue (Glubran2, GEM). During the same procedure, a pericardial drain was placed, and 650 mL of chylous fluid was removed.

TTE performed the next day demonstrated trivial residual pericardial effusion without echocardiographic features of tamponade. At 4- and 12-week follow-up, the patient remained asymptomatic without peripheral lymphedema, and repeat TTE demonstrated a small, stable effusion without significant reaccumulation.

## Discussion

Chylopericardium is an uncommon cause of pericardial effusion and is defined by the accumulation of chyle within the pericardial space.^1^ Chyle is a lipid-rich lymphatic fluid transported via the thoracic duct to the venous circulation. Disruption of this pathway or abnormal lymphatic-to-pericardial communication can result in chylous leakage into the pericardial sac.^1^^,^^2^ Chylopericardium may be primary (idiopathic) or secondary, with secondary causes reported more frequently.^1^ Secondary chylopericardium has been associated with thoracic trauma, cardiothoracic surgery, mediastinal malignancy, radiotherapy, subclavian venous thrombosis, congenital lymphatic disorders, infection such as tuberculosis, and inflammatory conditions including acute pancreatitis.^1^^,^^3^, ^4^, ^5^ Primary chylopericardium is a diagnosis of exclusion.

In a systematic review of 98 cases, Verma et al^6^ reported that chylopericardium predominantly affects younger patients (mean age: 37 years), most commonly presents with large pericardial effusions (80%), and is associated with cardiac tamponade in approximately 38% of cases. Yu et al^7^ examined 104 cases over 6 decades and found that nearly 40% of patients were asymptomatic at diagnosis despite large effusions on imaging. These findings suggest that significant chylous pericardial accumulation may occur insidiously, as observed in the present case. Diagnosis requires pericardial fluid analysis via pericardiocentesis or surgical drainage, with characteristics including triglyceride concentration >5.65 mmol/L, a cholesterol-to-triglyceride ratio of <1, and lymphocyte predominance.^6^ In this case, the pericardial fluid triglyceride concentration was 20.15 mmol/L, the cholesterol-to-triglyceride ratio was 0.1, and lymphocytes comprised 96% of cells. After laboratory confirmation, lymphatic imaging is used to delineate thoracic duct anatomy and localize chyle leakage. In the present case, imaging identified a network of lymphatic channels in the superior mediastinum and reflux from the thoracic duct, supporting a diagnosis of primary chylopericardium with a defined anatomic mechanism.

TTE is the first-line modality for detection, quantification, and hemodynamic assessment of pericardial effusions given its availability and low cost.^1^^,^^8^ Cross-sectional imaging with CT and MR provides a broader field of view, enabling assessment of loculated effusions, pericardial thickening or masses, and associated thoracic pathology.^1^^,^^8^ On CT, chylous pericardial effusions demonstrate low attenuation values, often approaching that of fat, reported between −60 and −80 Hounsfield units.^8^ Noninvasive lymphatic imaging (CT or MR lymphangiography or lymphoscintigraphy) localizes the leak and delineates lymphatic anatomy, which is crucial given the frequent variations in thoracic duct anatomy. In our patient, intranodal contrast-enhanced MR lymphangiography identified an abnormal signal from the upper thoracic duct, consistent with a leak into the pericardial sac. Additionally, it identified a solitary lower thoracic duct as an appropriate target for intervention. Compared with iodinated oil-based contrast agents used in conventional lymphangiography and lymphoscintigraphy, MR lymphangiography may offer higher contrast resolution, faster image acquisition, and improved visualization of smaller lymphatic channels given the lower viscosity of gadolinium.^9^ Across literature reviews, lymphangiography and lymphoscintigraphy were used in 59% to 63% of cases, whereas MR imaging was reported in as few as 10%.^6^^,^^7^ Given the limited number of reported cases, the sensitivity and specificity of each modality remains undefined and likely reflects local expertise and availability. In our patient, a stepwise approach based on advanced imaging was used to delineate thoracic duct anatomy and guide definitive therapy.

In hemodynamically stable patients, guidelines recommend initial medical management with dietary fat restriction using medium-chain triglycerides, total parenteral nutrition, and pharmacologic therapies aimed at reducing chyle production.^1^^,^^6^ Octreotide may be most beneficial in postoperative or malignancy-associated chylopericardium, but evidence is limited to case series.^1^^,^^6^ Urgent pericardiocentesis is indicated for patients with cardiac tamponade or significant symptoms to restore clinical stability.^1^ Medical therapy alone frequently fails, with up to 71% of cases ultimately requiring surgical intervention.^4^ In a systematic review of 18 primary cases, Dib et al^4^ reported a median time to surgery of 12 days (range, 1-1,460 days), suggesting that while most patients underwent early intervention, some experienced substantial delays. Surgical management is recommended for clinically significant disease, including daily pericardial drainage >1,500 mL, failure of drainage to decrease below 500 mL/d after 5 days, malnutrition, recurrent effusion within 3 months, and when a leak from the thoracic duct is identified.^1^^,^^4^^,^^7^ The increasing use of video-assisted thoracoscopic surgery reflects a shift toward less-invasive, targeted strategies.^4^^,^^7^ Pericardiectomy may be considered in select patients to prevent or manage constrictive physiology.^1^^,^^6^^,^^7^ This case demonstrates that percutaneous thoracic duct embolization is an effective, minimally invasive alternative to surgical thoracic duct ligation.

Mortality associated with chylopericardium is largely attributable to underlying comorbidities, particularly malignancy.^4^^,^^6^^,^^7^ Recurrent effusions are not uncommon, with one review reporting recurrence in approximately 16% of cases and rehospitalization in 10%.^6^ Recurrence is more frequent when pericardiocentesis or pericardial window is performed without thoracic duct ligation, with rates approaching 50% for a window alone, whereas recurrence after duct-directed interventions such as surgical ligation or percutaneous embolization is rare.^4^ This underscores the importance of identifying and treating the underlying lymphatic anomaly and maintaining appropriate postintervention surveillance imaging. This case highlights the evolving role of lymphatic imaging and percutaneous thoracic duct embolization as definitive therapy for idiopathic chylopericardium in contemporary cardiovascular practice.

## Conclusions

Recurrent primary chylopericardium may arise from a thoracic duct leak. Multimodality imaging, particularly MR and catheter-based lymphangiography, plays a key role in anatomic localization and treatment. Thoracic duct embolization can achieve durable resolution when pericardiocentesis alone is insufficient, offering a minimally invasive alternative to surgery.

## Funding Support and Author Disclosures

The authors have reported that they have no relationships relevant to the contents of this paper to disclose.

## Appendix

Video 1 Two-Dimensional Transthoracic Echocardiography Demonstrating Signs of Cardiac Tamponade Large circumferential pericardial effusion with right ventricular diastolic collapse.
